# Virtual Care and Health Care Access: Pragmatic Evaluation of Implementation, Acceptance, and Use in General Practice and Aged Care Homes

**DOI:** 10.2196/89019

**Published:** 2026-06-12

**Authors:** Adeola Bamgboje-Ayodele, Tamasha Jayawardena, Anita Higgins, Margaret Watkiss, Fiona Robinson, Susan Kurrle, Pan Teng, Melissa Baysari, Meredith Makeham

**Affiliations:** 1 Discipline of Design, School of Architecture, Design and Planning, The University of Sydney, 148 City Road, Darlington, 2008, Australia, 61 293519644; 2 Digital Health Human Factors Research Group, Sydney School of Nursing, Faculty of Medicine and Health, The University of Sydney, Camperdown, Australia; 3 Twilight Aged Care Homes, New South Wales, Australia; 4 Faculty of Medicine and Health, The University of Sydney, Camperdown, Australia; 5 Sydney North Health Network, New South Wales, Australia

**Keywords:** virtual care, implementation, acceptance, adoption, aged care, primary care

## Abstract

**Background:**

Health care access plays a central role in reducing inequities across populations. Virtual care can mitigate these inequities by facilitating more inclusive and accessible health care delivery. In residential aged care homes (RACHs), virtual care has the potential to enable timely and efficient access to general practitioners (GPs) for residents. However, as context, technologies, and users are complex, the implementation, acceptance, and use of virtual care technologies in RACHs remain challenging.

**Objective:**

This study aimed to evaluate the barriers and facilitators to implementing, accepting, and using virtual care technologies to connect residents with GPs for health care delivery in RACHs, and to identify benefits, unintended consequences, and opportunities for optimization.

**Methods:**

We conducted a pragmatic, cross-sectional qualitative study guided by interpretivist principles. Semistructured interviews were undertaken with 32 participants (11 GPs, 11 RACH nurses, 3 practice managers, 5 residents, and 2 carers). Data were analyzed inductively using reflexive thematic analysis and mapped deductively to the Systems Engineering Initiative for Patient Safety model to examine sociotechnical interactions influencing virtual care delivery.

**Results:**

Our investigation revealed that barriers to implementing, accepting, and using virtual care technologies to deliver care to RACH residents were more pervasive and salient in participants’ accounts than enablers. While barriers were found across all Systems Engineering Initiative for Patient Safety domains for GPs, most of the barriers for residents and carers were identified in the “people” and “organizational” domains, and in addition to these, “technology and tools” domain for RACH nurses. Although many barriers are common across the people (eg, resistance to using new technology), technology (eg, inadequate system integration), and organization (eg, logistical challenges) domains for RACH nurses and GPs, our study revealed unique barriers to virtual care delivery for residents and carers (eg, interruptions and potential to exclude residents from conversations) whose views are often absent from existing literature. Our findings also revealed that there is no standardized virtual care consultation process between RACHs and GPs—a key concern strongly associated with the identified work system barriers. While virtual care was seen as beneficial, participants identified some unintended consequences to patients (eg, loss of doctor-patient relationship), clinicians (eg, additional workload), and health care organizations (eg, infection control).

**Conclusions:**

Virtual care can improve access to timely, high-quality general practice services in RACHs, but its potential is constrained by sociotechnical, organizational, and workflow challenges. Addressing system integration, usability, staffing, training, and policy gaps, including funding and billing structures, will be crucial to realizing the benefits of virtual care. This study provides new evidence to inform design, implementation, and policy decisions supporting equitable virtual care delivery aligned with Sustainable Development Goals 3 and 10.

## Introduction

The world is aging [1]. As the needs of an aging population are severe and complex, the demand for health care access will continue to rise [2,3]. Health care access plays a central role in reducing disparities across populations. The 2030 Agenda for Sustainable Development Goals (SDGs), adopted by all United Nations members, identified health and well-being (Goal 3) and reduced inequalities (Goal 10) as key priorities [4]. With only 5 years to reach the SDGs, pervasive global disparities continue to undermine the equitable realization of these goals [5]. Virtual care technologies have transformative potential to reduce inequities in health care by making services more inclusive and accessible. However, without inclusive design, context-aware implementation, and fair scaling, they could unintentionally reinforce existing inequities.

Virtual models of care encompass a spectrum of modalities and technological complexity. These models may involve synchronous modalities (eg, telephone or video consultations) or asynchronous modalities (eg, email or text-based communication) [6], and may be supported by technologies ranging from relatively simple tools to complex, integrated systems such as remote monitoring devices, clinical decision support, and Bluetooth-enabled diagnostic instruments [7,8]. Many benefits of virtual care have been reported in primary care settings. For example, virtual care can improve primary care efficiency by facilitating the remote triage of patients, identifying those who require urgent face-to-face care from those who can be managed virtually [9,10]. In residential aged care homes (RACHs), existing research suggests that virtual care has the potential to enable timely and efficient access to primary care providers for residents [11]. While some evidence suggests that virtual care models can reduce avoidable hospitalizations [12] and subsequent health deterioration in residents due to shorter wait times [13], context, technologies, and users are complex, and there are many reports of challenges in adoption and intended outcomes not being achieved [13]. Additionally, adoption challenges associated with telephone consultations are likely to differ from those associated with video-enabled consultations requiring peripheral devices, clinical system integration, and staff facilitation. In a previous review of 26 articles describing the experiences and perspectives of health care providers involved in virtual care services in RACHs, usability problems emerged as a key barrier to uptake [14]. This has also been identified as an issue for residents using virtual care services [15]. In addition to poor usability, barriers to virtual care uptake include end user factors (eg, hearing difficulties) and implementation factors (eg, using multiple platforms and poor workflow integration) [15,16]. Our more recent systematic review of 13 articles on the barriers, enablers, processes, and outcomes of virtual care delivery in RACH settings echoes these findings and indicates limited evidence on residents’ and carers’ perspectives [17]. These studies highlight the importance of virtual care aligning with residents’ and health care providers’ preferences and capacity, and the significance of considering the complex contexts of their use in a technology implementation (ie, the sociotechnical work system) [16].

Despite significant efforts to optimize the implementation, acceptance, and use of virtual care technologies in RACHs, these remain an ongoing challenge. Limited comprehensive evaluations that provide in-depth insights into the various sociotechnical factors that enable successful virtual care implementation in RACH settings have been conducted. While challenges related to usability, workflow integration, and system interoperability have been documented following existing evaluations [17], far less is known about how virtual care is experienced by residents of aged care homes, particularly in relation to communication, involvement in care, and the doctor-patient relationship.

This study aimed to evaluate the implementation, acceptance, and use of virtual care technologies used in RACHs to connect residents with general practitioners (GPs) for health care delivery. In this study, we conceptualize virtual care as encompassing synchronous and asynchronous consultations supported by varying levels of technological complexity. Our investigation focused on three key questions: (1) what are the barriers and enablers to implementing, accepting, and using virtual care technologies to deliver care to RACH residents from the perspective of GPs and their teams, RACH residents (or their carers), and RACH staff? (2) What are the benefits and unintended consequences of virtual care implementation on residents, clinicians, and health service organizations? And (3) How might we optimize the implementation, acceptance, and use of virtual models of care from the perspective of GPs and their teams, RACH residents (or their carers), and RACH staff?

## Methods

### Study Design

The study was informed by a pragmatic qualitative orientation as we focused on understanding real-world implementation challenges and informing practice improvements. Additionally, our study was guided by interpretivist principles to explore participant experiences. Accordingly, we conducted a cross-sectional qualitative study, which is a design appropriate for providing insights into people’s experiences of a complex phenomenon or activity [18]. We conducted semistructured interviews to explore participants’ experiences. The COREQ (Consolidated Criteria for Reporting Qualitative Research) checklist was used to report the study (Checklist 1).

### The Virtual Care Platform and Setting

The suite of virtual care technologies included a platform with videoconferencing functions, secure document and image sharing, a 6-in-1 remote monitoring device for measuring vital signs (eg, temperature and oxygenation), and other remote technologies that connected to the 6-in-1 device via Bluetooth technology (eg, digital stethoscope, otoscope, throat scope, and urine analyzer). The virtual care platform also provides integration with some RACH and GP clinical information systems.

The suite of virtual care technologies was implemented at the Sydney North Health Network (SNHN), which spans a large geographic area (approximately 900 km^2^ covering 9 local government areas). It included 103 RACHs with around 9000 beds, and 288 general practices with 756 GPs. The virtual care service was implemented to improve the level of service from GPs to RACHs. Across the RACHs that opted to use the service, SNHN provided a suite of hardware, software, and training in 2024. For this study, we included those who had implemented any type of virtual care technology, including the SNHN virtual care platform.

In Australia, GPs providing care to residents of residential aged care homes are predominantly funded through the national universal health insurance scheme, Medicare, via fee-for-service payments under the Medicare Benefits Schedule (MBS). These arrangements cover both face-to-face and telehealth consultations, including services delivered to residents in aged care settings. During the study period (2024‐2025), telehealth consultations were a permanent feature of general practice funding in Australia.

### Participant Recruitment

GP participants and RACHs were invited via the SNHN Newsletter, flyers, and targeted email invitations from SNHN. All RACHs who had engaged with a virtual care technology and all GPs across SNHN who provide services to these homes were invited to participate. General practice participants (GPs and practice managers) were invited via letters delivered by post. The managers at the RACH sites were asked to nominate staff who were involved in, or interested in, supporting a virtual care consult between a resident and GP, and these staff were invited to participate. RACH managers were also asked to identify potential residents who satisfy eligibility criteria for researchers to invite into the study. Eligibility criteria for our participant groups (ie, GPs, RACH staff, practice managers, residents, and their carers) are shown in Table 1.

**Table 1.** Eligibility criteria for the 5 participant groups.

Participant group	Eligibility criteria
GP^a^	Current Australian Health Practitioner Regulation Agency registration as Specialist General Practitioners Current or previous experience with visiting RACHs^b^ to provide care for residents
RACH staff	Working in a RACH in the SNHN^c^ region that implemented a virtual care platform for conducting consultations with GPs
Practice manager	Working in the general practices of consenting GPs
Resident	Able to read, understand, and speak English Able to provide informed consent Aged 65 years or more Had no severe cognitive function restrictions (eg, established dementia) Identified as suitable by their RACH manager and usual nursing staff, based upon their clinical judgment
Carer	Invited by their RACH resident participant and Could read, understand, and speak English

a GP: general practitioner.

b RACH: residential aged care home.

c SNHN: Sydney North Health Network.

### Data Collection Procedure

Data were collected from September 2024 to April 2025, when thematic saturation occurred. A semistructured interview guide was developed by the research team with expertise in qualitative research, human factors, implementation science, psychology, information technology, medicine, and nursing (refer to Multimedia Appendix 1 for the guide). The interview guide was piloted with clinician-researchers who were members of the broader team, and the guide was subsequently refined for clarity. All interviews were conducted in person or online via videoconference and audioconference, recorded and transcribed verbatim. Interviews were led by an experienced qualitative researcher (AB-A) or trained assistant (TJ), neither of whom had prior ties to the organizations. Data collection continued until thematic saturation was reached across the overall dataset, particularly for the primary stakeholder groups (GPs and RACH staff), recognizing that saturation was not sought or achieved for all participant subgroups individually.

### Data Analysis

Demographic data were analyzed descriptively using Microsoft Excel. For the qualitative data, our analytical approach included data familiarization, coding, generating initial themes, reviewing potential themes, defining and naming themes, and producing the manuscript. Specifically, 2 researchers (AB-A and TJ) familiarized themselves with the transcribed data by reading it multiple times before independent coding. Interviews were inductively analyzed using NVivo software (Lumivero) [19]. Two researchers (AB-A and TJ) independently coded the first interview transcript and met to discuss and reach consensus on codes. After initial code generation, the researchers developed themes by merging codes with a shared meaning. The remaining interviews were coded by one researcher (AB-A) using the coding framework agreed upon. Following this, the researchers reviewed the themes to ensure coherent patterns were formed, which contributed to the overall narrative and interpretation of the data. Researchers met periodically throughout data collection to discuss the interviews (AB-A and TJ).

After inductive coding, themes from interviews were deductively mapped to elements of the Systems Engineering Initiative for Patient Safety (SEIPS) model by AB-A and TJ in a 3-hour workshop. The SEIPS model focuses on how people, tasks, technologies and tools, organizational factors, and environments interact to shape care processes and outcomes [20]. Specifically, we adopted the SEIPS model to examine virtual care because it explicitly conceptualizes technology use and acceptance as outcomes of interactions within a sociotechnical work system. This perspective is particularly well-suited to residential aged care settings, where virtual care delivery is highly mediated by staff roles, workflow coordination, organizational policies, and infrastructure. By using SEIPS, we position acceptance and use of virtual care not as purely individual psychological phenomena, but as emergent properties of work system design and implementation. This enables a more comprehensive examination of barriers, enablers, unintended consequences, and opportunities for optimization across multiple stakeholder groups.

Following this, AB-A presented the codes and themes to other members of the team (MM and MB) for further refinement until consensus was reached in a 2-hour workshop. Results are presented based on the SEIPS work system elements, including people, tasks, tools, organization, and environment, identifying barriers, facilitators, and elements with dual traits (ie, viewed as both a facilitator and a barrier).

In qualitative research, 4 key criteria: credibility, confirmability, dependability, and transferability, are commonly used to strengthen the rigor and trustworthiness of a study. To enhance credibility, most (20/32) interview sessions were led by a senior researcher (AB-A) with substantial experience in digital health, virtual care, and qualitative research. The senior researcher trained, supervised, and/or was present in the remaining interviews conducted by a junior researcher (TJ). To ensure confirmability, we adopted an investigator-triangulation approach. Multiple members of the research team collaboratively reviewed themes in a 2-hour workshop and brought diverse perspectives to the interpretation of the data. Dependability was supported through the development of a detailed study protocol, drafted collaboratively by the research team in consultation with relevant stakeholders. We also ensured coding reliability by recording, transcribing, and carefully reviewing all audio data. To promote transferability, we used purposive sampling to recruit participants who represented the full range of stakeholder groups relevant to the project. We aimed to provide rich contextual information about these groups; however, identifying details were removed from all transcripts and quotations to protect anonymity and ensure that individual stakeholders could not be recognized.

### Ethical Considerations

Ethics approval was obtained from the University of Sydney’s Human Research Ethics Committee (HE000483). Written informed consent was obtained from all participants, who were informed of their right to withdraw from the study at any time. Participation was voluntary, with gift cards offered as compensation for their time. All identifying characteristics have been removed to protect anonymity.

## Results

### Demographic Characteristics

Individual semistructured interviews were conducted with 32 participants and lasted 25 minutes on average (total: 801 min). A list of the participants interviewed and their roles is provided in Tables2  3.

**Table 2.** Participant demographics for the GP^a^, nurse, and practice manager groups.

Participant type	Number	Age (years), mean (SD; range)	Sex, or sex assigned at birth, n (%)	Participants who have used audio-based telehealth (n)	Participants who have used video-based telehealth (n)
GP	11	42 (7.8; 25‐54)	Female: 7 (63.6) Male: 4 (36.4)	11	2
RACH^b^ nurse	11	48 (9.2; 35‐55)	Female: 11 (100)	8	5
Practice manager^c^	3	43 (5.7, 35‐54)	Female: 3 (100)	—^d^	—

a GP: general practitioner.

b RACH: residential aged care home.

c One practice staff had a dual role as a practice nurse and a practice manager.

d Not reported.

**Table 3.** Participant demographics for the resident and carer groups.

Participant type	Number	Age (years), mean (SD; range)	Sex, or sex assigned at birth, n (%)	Resident’s LOS^a^ at RACH^b^, duration (months), mean (SD; range)	Audio-based telehealth, n (%)	Video-based telehealth, n (%)
Resident	5	88 (3.9; 82‐91)	Female: 3 (60) Male: 2 (40)	11 (8.2; 3‐36)	3 (60)	2 (40)
Carer	2	79 (7.7; 73‐84)	Female: 1 (50) Male: 1 (50)	24 (6; 12‐36)	1 (50)	0 (0)

a LOS: length of stay.

b RACH: residential aged care home.

All participating GPs were in practices that had a manager and a nurse on site. Most GPs serviced only one RACH (n=6), while some serviced more than one (n=3), with one servicing 20 RACHs. Insights from participants were either specific to the SNHN Virtual Care platform (with 4 RACH nurses trained to use the platform) or generally on virtual care technologies. At the general practices where our GP participants were based, the number of RACH patients serviced ranged from 25 to 70. All residents who took part in the interviews reported that their GPs had previously visited them at their RACH. Only one carer had experienced GP visits and telephone consultations together with their resident (refer to Multimedia Appendix 2 for further demographic details).

### Work System Elements

**Figure 1.**
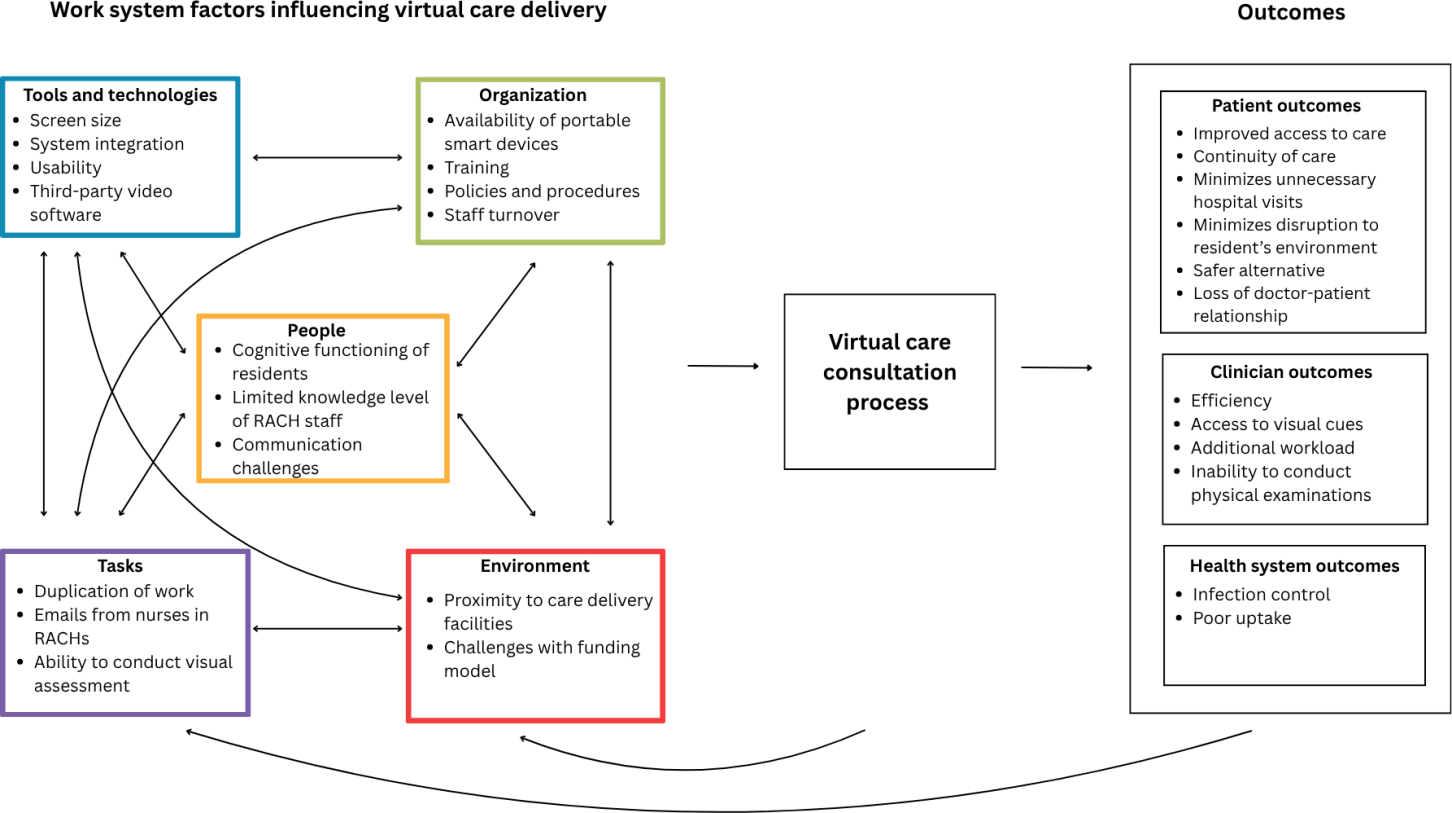
An overview of themes mapped to the Systems Engineering Initiative for Patient Safety model. RACH: residential aged care home.

An overview of how the themes align with the SEIPS model is shown in Figure 1, and a list of themes, subthemes, and illustrative participant quotes appears in Multimedia Appendix 3.

Different participant groups identified varying barriers and facilitators, with some SEIPS domains of more concern to some groups than others. A summary of the perceptions of the participant groups concerning the barriers and facilitators to virtual care delivery is shown in Table 4.

**Table 4.** Perceptions of participant groups mapped to the SEIPS^a^ domains^b^.

SEIPS domain and perceptions (+ enablers, – barriers)	RACH^c^ staff	Primary care providers	Residents and carers
People			
Cognitive functioning of residents –	✓		
Limited knowledge level of RACH staff –	✓	✓	
Resistance to using new technology –	✓	✓	
GP^d^ preference and willingness –	✓	✓	
Digital literacy ±		✓	
Communication challenges –			✓
Potential to exclude residents from conversations –			✓
Familiarity between the resident, RACH nurse, and GP ±			✓
Task			
Duplication of work –	✓		
Emails from nurses in RACHs pass along the burden of responsibility –		✓	
Inefficiency –		✓	
Ability to conduct visual assessment +		✓	
Ability to collect and share vital signs data +		✓	
Interruptions –			✓
Technology			
Screen size ±	✓	✓	
Lack of system integration –	✓	✓	
Poor usability –	✓	✓	
Poor network connectivity –	✓	✓	
Technology glitches –	✓		
Inability to conduct full assessment –		✓	
Difficulties accessing clinical information systems –		✓	
Third-party video software +		✓	
Remote access to clinical information systems +		✓	
Organization			
Awareness of virtual care options –	✓		
Availability of portable smart devices –	✓		
Training –	✓		
Management of privacy –	✓	✓	✓
Logistical challenges –	✓	✓	
Organizational policies and procedures –	✓	✓	
Availability of registered nurses during the virtual consult +	✓	✓	
Limited working hours –		✓	
Staff turnover –		✓	
Inadequate RACH staff training –			✓
Environment			
Proximity to care delivery facility –		✓	
Challenges with the funding model –		✓	

a SEIPS: Systems Engineering Initiative for Patient Safety.

b Legend: (–) indicates barrier, (+) indicates enabler, and (±) indicates both barrier and enabler.

c RACH: residential aged care home.

d GP: general practitioner.

### People Domain

The complexity of a resident’s care needs were reported by RACH nurses as a key factor influencing health care providers’ decisions on whether a virtual or face-to-face consult was appropriate. Simple care needs with clear and well-defined problems were viewed as suitable for virtual care consultations, whereas complex care needs requiring investigations were said to be more attuned to face-to-face consultations. RACH nurses explained that patients with mental health challenges or those requiring end-of-life discussions or advanced care planning would benefit more from in-person consults.

*...end of life care is something that I think you need, that personal touch is very important...*

[P2, registered nurse]

Digital literacy levels of RACH nurses and residents were viewed by GPs as either an enabler (high level) or a barrier (low level) to visual assessments in virtual care consultations. Familiarity between the resident, RACH nurse, and GP was reported by nurses and residents as an enabler of virtual care consultations, which was seen to result in trust and a meaningful relationship being built over time. However, lack of familiarity was said to worsen the virtual care experience. Good cognitive functioning in residents enabled the acceptance of videoconferencing from the perspective of RACH nurses, whereas residents with cognitive decline were said to be hesitant, resistant, and unable to understand the virtual care consultation.

*...it could be bit challenging, and sometimes residents with dementia here, they may not understand the process, and they are more resistant and hesitated*

[P12, registered nurse]

Communication challenges were identified as barriers to virtual care, with residents reporting that these arose from hearing impairments and language differences between the resident, RACH nurse, and GP, often resulting in information being lost in translation. Additionally, both residents and carers reported that RACH residents lose their autonomy and direct access to their GP, and this concern could be further exacerbated by virtual consultations where the nurse oversees the consultation resulting in the potential to exclude residents from conversations pertaining to them.

*He does everything I want on the phone and not direct with me, just the nurse. I tell the nurse, yeah, and she goes I will talk to him. She comes back and interprets whatever he says. I’ve never seen him. I don’t know if he exists.*

[P29, resident]

Participants’ accounts suggested that resident exclusion during virtual consultations occurred through interacting factors rather than a single mechanism. For example, the physical positioning and handling of the device by the RACH nurse, combined with small screen sizes and audio limitations, could limit residents’ ability to see or hear the GP clearly. These constraints were compounded for residents with hearing impairment or language differences, particularly when GPs and nurses communicated primarily with each other to progress the consultation efficiently.

Furthermore, limited virtual care knowledge levels of RACH staff were flagged by GPs and RACH nurses as another barrier to virtual care consultations, which participants noted could lead to information being passed on incorrectly, resulting in confusion. Also, resistance to new technology was reported by RACH nurses and GP participants as they explained that older GPs were perceived to have a negative perception of virtual care, which was viewed to have stemmed from previous experiences with technologies. Similarly, some RACH nurse participants were of the opinion that some GPs preferred in-person visits to virtual visits, as they believed these GPs lacked the willingness to learn new technologies.

*...it’s so frustrating to see that like us as GP...we're really resistant to change...*

[P4, general practitioner]

### Task Domain

RACH nurses explained that the tasks that triggered either phone or video consultation requests to GPs included wound and skin care, medication management, palliative care, case conferences, check-ups, and determining treatment plans, particularly for residents with low to medium acuity levels. Once any of these tasks triggered the need for a consultation, it was stated that the nurses then progressed to preconsultation tasks. Appointment booking was said to occur via phone calls, emails, and, on rare occasions, text messages from the RACH to the practice. The approach to booking was perceived to be determined by the urgency of the resident’s concern or GP-specific practices, which might involve nurses calling the GP’s practice within a specified time. Following appointment booking, it was mentioned that the nurse may be asked to collect some preconsultation observations, for example, blood pressure, urinalysis, and blood oxygen saturation. However, it was reported that this task (ie, collecting vital signs data) may not be performed unless it is specifically requested by the GP prior to the consult. From the GP’s perspective, a task sometimes performed prior to consultations was the review of the resident’s record. Documentation was reported to be the major postconsultation task that is completed by either or both the nurse and the GP. It was further mentioned that some documentation tasks following a virtual care consult were done via email between the GP and the RACH nurse.

*...if it’s basically weekend, or if it’s basically, I am out of Sydney, so I don’t have access to my own PC at home to get access to the remote access to the Best Practice. Then I will get nurse to write on my behalf…by the time that I get to the telehealth or to the Sydney I have access either to my remote control at home or at the practice, I’ll do my notes as well*.

[P18, general practitioner]

Barriers to appointment booking, consultation, and documentation tasks were reported. For appointment booking, GP participants explained that emails from RACH nurses pass along the burden of responsibility to GPs, which can both be challenging and consequential, resulting in delayed patient care. Participants noted that this task was experienced as burdensome in the context of existing organizational workflows and funding arrangements. This led to emails being discouraged in general practices as a communication approach with RACHs, with GP participants suggesting that RACHs should be responsible for ensuring care continuity. However, RACH nurses reported that emails are preferred as it fulfills the requirement for documentation. The main barrier to consultation was reported to be interruptions as this was flagged as a concern from the resident’s perspective.

*...he doesn’t ever turn his things off properly. Even when we’re in the room, there’ll be people you know in the room or you can hear the click of his phone that he’s got in front of him… Apparently they [other clinicians] aren’t allowed to do some things without his authorisation. So rather than hold them up all the time, they come in and interrupt you as you are talking…And he tended to be combining sometimes, dealing with me and dealing with something coming over the computer...*

[P31, resident]

For documentation tasks, inefficiency was highlighted as a concern, as GPs noted that it takes too much time to document and upload their notes into their own clinical information systems following a virtual consult. RACH nurses also added that the burden of documentation stemmed from mandatory reporting requirements, which were further exacerbated by duplication of work (ie, documenting the same information multiple times in multiple places).

*When you’ve got to repeat what you’ve done potentially three or four times, you know, whether you’re a PDF document or you cut and paste, it’s a real issue...*

[P6, registered nurse]

Enablers of virtual care consults were perceived to include the ability to conduct a visual assessment as opposed to a telehealth (audio-only) consult from the perspective of GPs. Also, GP participants highlighted that the ability to collect and share the vital signs data between the RACH and the GP facilitates improved virtual care delivery.

*I think the biggest thing is always just, it’s just being able to see the patient. I think that’s the main thing. To be able to see them and talk to them and see what they’re doing is a massive deal.*

[P3, general practitioner]

### Technology and Tool Domain

Within this domain, GPs and RACH nurse participants described a range of technologies and tools that supported virtual care delivery, as well as facilitators and barriers to the use of those technologies. Four types of technologies were described that supported or enabled virtual care, including videoconferencing platforms (eg, Zoom [Zoom Communications, Inc], FaceTime [Apple Inc], Coviu [Coviu Global Pty Ltd], and WhatsApp [Meta Platforms, Inc]), remote monitoring technologies (eg, digital stethoscope and pulse oximeter), clinical information systems (eg, electronic medication record and electronic patient record), and communication technologies (eg, email and text message). RACH nurses reported that paper-based medication charts were the only tool supporting virtual consults.

A large number of barriers and facilitators relating to technologies and tools were mentioned in interviews. Screen size was viewed to either enable (if larger) or impede (if smaller) visual assessments in virtual care consultations from the perspective of both GPs and RACH nurses. The most frequently reported barriers were lack of system integration, poor usability, and poor network connectivity. Lack of system integration was highlighted as the key source of data fragmentation in clinical information systems, with GP participants reporting that this makes documentation and continuity of care difficult to achieve in a seamless manner. Within RACH settings, GP participants further noted that different patient record systems were in place in different homes, and RACHs were using a national medication system that hospitals could not access, making continuity of care challenging. Poor usability was seen to be another barrier to conducting virtual care consultations, with both GP and RACH nurse participants emphasizing that some virtual care technologies are not user-friendly, not easy to learn, and that infrequent use meant that users were not able to remember how to navigate them, resulting in difficulties in setting up the technology. Poor network connectivity was identified as another problem, with GP participants explaining that it could impact the video and the audio quality of a virtual care consult, resulting in the need to terminate such calls and, on occasion, opting for high-resolution photos shared by text messaging services.

*...good internet, so enough bandwidth to facilitate a good, clear call, you know, without it being choppy. And so that’s number one. And then also, you know, that affects audio. It affects the clarity of the video. You know, if I’m doing a telehealth review of the wound, if I get really grainy, terrible video, it’s of no value… So sometimes, in those cases, I will then get the RN to send me a high-resolution photo via text, okay, just to back up that kind of review of the wound.*

[P17, general practitioner]

The reported enablers of virtual care relating to technology and tools were the use of third-party video software and remote access to clinical information systems. GP participants explained that third-party video software such as Apple’s FaceTime and Meta’s WhatsApp, which are readily available on smart devices, was preferred to purpose-built videoconferencing software, as they were easy to use and saved time. Additionally, remote access to clinical information systems enabled and simplified documentation tasks associated with each consultation, as stated by practice managers and RACH nurses. Also, access to these clinical information systems was viewed by GP participants as a facilitator to prescribing medications and a means of having visibility over the patient’s trajectory. Although different RACHs have different systems with varying levels of access to clinical information systems for GPs, instances where GPs have remote access to RACH systems were viewed positively.

*...so I could, wherever I am, actually look at the notes that if they’ve done something, I can see. Also the prescription, the medication chart was also online as well, so it’s easy when I need to prescribe something...*

[P4, general practitioner]

### Organization Domain

Awareness of virtual care options, availability of portable smart devices, training, and management of privacy were all seen to both impede and facilitate effective virtual care delivery. RACH nurse participants noted that it was imperative to educate residents and their families about the virtual care options available to increase awareness. Conversely, when consumers lacked awareness of the available options, RACH nurses explained that they tended to choose emergency department visits, impeding virtual care delivery. Additionally, RACH nurses said that the availability of portable smart devices enabled virtual care calls due to the ease of moving the camera of such devices (eg, smartphone) during a virtual care consultation when compared to a much bigger device (eg, computer). However, RACH nurses also stated the lack of portable smart devices resulted in them using their own personal devices to facilitate the virtual care call, highlighting the lack of organization-issued portable smart devices to support virtual care consultations.

*...at the moment, if we do have to have a telehealth, it needs to be done by our own private phone, which is not really ideal. We don’t have anything in place at this stage… [we need RACH organisations to] buy a device for the nurses, because we need [it]...*

[P16, registered nurse]

Training was perceived as both enabling and hindering virtual care consultations: RACH nurses felt it helped GPs and nurses learn to use the system, while residents viewed it as inadequate. It was further reported by RACH nurses that virtual care consultation training should be extended to care staff in RACHs, as there are instances when nurses are unavailable to support the call, and participants noted that inadequate staff training results in poor resident experience during the virtual care consult. In addition, how a resident’s privacy was managed during videoconferencing could either enable the consult when conducted in the resident’s own room (if not shared) or impede the consult in cases where residents shared rooms. GPs expressed that maintaining privacy for residents was often not feasible, particularly when cognitive impairments meant nursing staff had to be present during consultations. Although third-party software was popular for virtual care, GPs and RACH nurses noted privacy concerns when using personal devices, as it risked sharing their mobile numbers without consent.

*...FaceTime would be more effective, but I don’t use that because it gives my phone number to the person, which I don’t want to do...*

[P20, general practitioner]

The most frequently reported organization-level barriers to virtual care consultations were logistical challenges and organizational policies and procedures. Logistical challenges in organizing virtual care calls often impeded consultations. GP and RACH nurse participants highlighted the absence of a clear scheduling structure, with difficulties in aligning the right people at the right time. These issues persisted across both video and audio modalities. Organizational policies also created barriers: some RACHs required staff to transfer residents to the hospital after any incident, regardless of severity, limiting opportunities for virtual care. GP participants further noted that policy development has lagged behind the implementation of virtual care technologies.

*...an issue is the company policies on various issues that happens or incidents that happen. We have policies that mandate that we have to transfer to people to hospital to be investigated…so that’s something to think [about] and each organisation has different policies and procedures around transfer to hospital...*

[P2, registered nurse]

The availability of registered nurses during the virtual consult was identified by GP participants as an important organizational enabler to their ability to undertake a virtual care consultation with a resident.

### Environment Domain

A total of 3 unique barriers to virtual care delivery were identified in this domain: 2 in the internal environment and 1 in the external environment. Within the internal environment, practice managers and GP participants reported that virtual care was usually not necessary as they were physically located in proximity to the RACH. Noise was seen as another barrier to virtual care consults, as practice manager participants noted that the presence of noise during the call could hamper communication.

*the voice [was] not clear due to the background noise...*

[P10, practice manager]

Within the external environment, a challenge with the funding model was flagged as a deterrent to conducting virtual care in RACHs. GP participants reported that the process of receiving payments was often laborious and frustrating, and that the requirement to always have the client on the call is not realistic. This barrier was said to have resulted in unpaid work or GPs choosing to abandon virtual care as a means of conducting consultations with RACH residents altogether.

*So there’s still a lot of unpaid work being done at my nursing home...which is really like frustrating.*

[P3, general practitioner]

### Virtual Care Consultation Process

RACH nurse participants reported that there was no standard way of conducting virtual care consultations between RACHs and GPs. They also noted varying approaches to conducting a virtual care consultation with differences occurring based on urgency, capacity of the resident, availability of a registered nurse, technologies available, time of day, and other work system factors. It was also highlighted by GP participants that nurses in RACHs often sought informal consultations from GPs about residents via text messages and emails without booking appointments. This was sometimes viewed by GPs as unpaid work due to the difficulty in billing processes for informal consultations that were not booked appointments directly involving the resident.

*We don’t have a process as such...We don’t have that. I have never even looked into if there is a policy of the video call, what exactly you’re supposed to do.*

[P16, registered nurse]

### Outcomes

Within this domain, participants reported positive and negative outcomes of virtual care delivery for residents, clinicians, and health services. For residents, the most significant benefit GP participants identified was improved access to care for residents in RACHs. Other benefits noted by RACH nurses and GPs included reduced unnecessary hospital visits and fewer disruptions to residents’ familiar environment. However, the potential loss of the doctor-patient relationship was flagged by GP participants as an unintended consequence of virtual care delivery in RACHs.

*There’s something that happens when you’re in a room with someone...it’s just a human-to-human interaction. Yeah, there’s something that it’s just lost ... you know that patient-doctor relationship, there is something about...meeting people, safe in their hand.*

[P17, general practitioner]

GP participants stated that virtual care delivery saved clinicians travel time, which enabled faster access to care for more residents. However, they also highlighted some inefficiencies due to the lack of integration of clinical information systems and logistical challenges. Additionally, GPs mentioned access to visual cues as a benefit of virtual care consultations with video. Both GP and RACH nurse participants stated that the unintended consequences of virtual care delivery for clinicians were additional workload and the inability to conduct physical examinations.

*And there’s other things... they’ve got to do with assessment and maintaining the aged care quality standards ... that keeps them time poor ... because they’re trying to do this paperwork. And, I think those reasons are the reason they see this as [extra workload] ...the impact of this on our already busy life, or work life, does put a strain on them…and the workload. The workload is huge.*

[P6, registered nurse]

For health services, RACH nurses explained that virtual care consultations minimize the spread of infections (as seen during the COVID-19 pandemic) but simultaneously create the need for additional infection control procedures in RACHs, for example, disinfecting devices like tablets and phones. Poor uptake of virtual care was viewed by GPs as an unintended consequence of poor implementation in RACHs, with many reports of virtual care technologies not being used and resulting in desired outcomes not being achieved. When nurses were asked how often virtual care calls were conducted in their RACHs, many reported that it was very infrequent.

### Recommendations

Participants provided some recommendations for improving virtual care delivery between RACHs and general practices, as shown in Table 5.

**Table 5.** Recommendations for improving virtual care delivery.

Theme and code	Quote
External environment	
Improve funding model	“I think that [NDIS funding] model is much better suited because a lot of the Medicare rules actually just don’t work for nursing home patients, especially with the patients with dementia. Technically, if I bill for 20 minutes of my time, I have to have spent all that 20 minutes with you, not talking to the nurse or… And actually, Medicare says that all that doesn’t count … So it is very not compatible. And secondly, it doesn’t allow you to collaborate with other people, where NDIS does that all the time, … I, as a GP, cannot speak to a cardiologist about your care and bill for that… I can’t bill the cardiologist for taking my time, and I can’t bill you” (P13, GP)
Organization-focused implementation strategies	
Create efficient process for video calls in RACHs^a^	“So having that ability, that knowing that … you can still do that telehealth conference without having to first call them and say, Hey, I’m about to call you on the program” (P15, GP)
Provide more realistic and ongoing training	“So I think telehealth is a great intervention. Maybe more training, doctors need to try it out for themselves and to be with the patients and see what does work for them and what doesn’t” (P31, resident)
Broaden population of staff who receive training	“Although it would be good if my nurse got trained…because my nurse could help with some of it too, and she could set up, yeah, she could almost get it ready for me, and so that I’m not wasting my time waiting for them” (P3, GP)
Provide dedicated smart devices in RACHs	“there needs to be a dedicated phone that the doctor can call and arrange an appointment with it” (P3, GP)
Provide stepwise resident onboarding	“introduce this stuff to residents little bit at a time, given the reason why it’s happening, present it at their meetings. Tell them where we’re going to use it, why we’re going to use it. Ask them why they think that’s not going to be the right thing to do, because it’s really just the doctors that are going to be at the other end of the machine” (P7, RN)
Promote virtual care via success stories	“if you have a nursing home that seems to have a very smooth use of this and an efficient use of this, then often one person showing another person how it’s good” (P4, GP)
Use word of mouth approach to promote virtual care	“I think part of it maybe, is probably with no word of mouth, actually, like one GP telling another GP” (P4, GP)
Technology-focused strategies	
Provide system integration	“I think virtual care would be amazing if they can start to logistically integrate everything into the one database that people can access” (P5, GP)
Provide an integrated app for virtual care	“Can we have national thing? Can we have an app we can download… I just think if the Department of Health, they can start an app just to facilitate that part of the consultation, that would be cool” (P1, RN) “If I had an app that could be on my phone, like, theoretically, they could set up a consult with all the obs like that would be ideal if they said, here’s your consult coming in, and then I take it … almost like an Uber Doctor consult” (P3, GP)
Provide remote monitoring devices	“I think a [digital] stethoscope would be one of the most useful things, because I feel as though with, you know, with guidance from a very senior clinician, you could probably direct a relatively well skilled nurse, to elicit the information that you require, just through placement…I would love to utilise those types of things, because I think that is useful” (P27, RN)
Ensure technology usability	“I guess the set-up steps needs to be very specific, like very detailed. It shouldn’t be a lot of steps… So it needs to be very simple, like telephone simple, like they only need to click a few buttons to get connected…Because it’s kind of like a big difference from Zoom or Teams like, you have to go into a waiting room until you got connected” (P9, RN)
Consider providing real-time language translation	“Heidi [platform] and all are working on it, the ability to translate in real time would be really good*”* (P13, GP)
Provide 3D rotation technology	“some type of 3d rotation thing with a picture, [to] try to take picture of some ulcer, or some lesion” (P11, GP)

a RACH: residential aged care home.

## Discussion

### Principal Findings

In this study, we sought to evaluate the implementation, acceptance, and use of virtual care technologies used in RACHs to connect residents with general practitioners for health care delivery. Our investigation revealed that barriers to implementing, accepting, and using virtual care technologies to deliver care to RACH residents were more pervasive and salient in participants’ accounts than enablers. While barriers were found across all SEIPS domains for primary care providers, we mostly identified barriers for residents and carers in the “people” and “organizational” domains, and in the same domains, including “technology and tools” for RACH staff. Although many barriers are common across people (eg, resistance to using new technology), technology (eg, lack of system integration), and organization (eg, logistical challenges) domains for RACH staff and primary care providers, our study revealed unique barriers to virtual care delivery from the perspective of residents and carers (eg, interruptions, potential to exclude residents from conversations). This provides an important perspective, as the views of residents and their carers are largely absent from the existing literature describing RACH and general practice virtual care. Our findings also revealed that there is no standardized virtual care consultation process between RACHs and GPs. This was a key concern strongly associated with the identified work system barriers (eg, logistical challenges). Additionally, we found that virtual care delivery in RACH settings was largely beneficial to patients, but we also identified some unintended negative consequences, including loss of doctor-patient relationships, additional workload for clinicians, and impacts on health care organizations (increased requirements for infection control)—which warrant further investigation.

Our findings shed light on residents’ perceptions of virtual care delivery, noting interruptions, potential to exclude residents from conversations, communication challenges, inadequate RACH staff training, and poor management of privacy as key barriers. Our recent systematic review of 13 articles on the barriers, enablers, processes, and outcomes of virtual care delivery in RACHs and primary care identified only one article providing insights on residents’ views [17]. The article explored residents’ views on ethical challenges associated with the use of virtual care and identified communication barriers and privacy concerns, aligning with our findings [21]. From the residents’ perspectives, our study highlights 2 novel findings: interruptions to GPs during virtual consultations and the risk of residents being excluded from virtual care conversations. These novel barriers to virtual care delivery can negatively impact residents’ experiences and can contribute to the loss of the doctor-patient relationship, which was identified as an unintended consequence. Viewed through a sociotechnical lens, our findings illustrate how interactions between tools (device form factor and audio-visual quality), people (residents’ sensory and cognitive capacities), and tasks (clinically focused communication between staff) can unintentionally marginalize residents within virtual care encounters, despite the technology being introduced to improve access. These findings also raise ethical considerations regarding how residents are positioned within virtual care encounters. Participants’ accounts suggest that virtual care may risk marginalizing residents’ experiential knowledge and involvement in decision-making. Such interactional shifts help explain participants’ perceptions of a loss of the doctor-patient relationship and highlight the importance of designing virtual care models that actively support residents’ participation to maintain person-centered care.

Consistent with our study findings, inadequate RACH staff training is a well-documented barrier to virtual care delivery in the literature [17]. Although existing studies have recommended effective and regular training on virtual care technologies in RACH settings [22,23], our findings were aligned with other studies that highlighted the barriers to achieving this due to the unique characteristics of this setting (eg, high workload, high staff turnover rates, and low digital literacy) [11,22,24-27]. These factors make it challenging to rely on training, and there have been calls for virtual care technologies in RACH settings to be easy to learn [28].

This study highlighted that no standardized virtual care consultation process currently exists between RACHs and GPs within the context investigated. This finding aligns with our existing systematic review that found that none of the 13 articles reviewed provided details on their virtual care consultation process [17]. Previous studies have clearly identified how virtual care is performed using process maps in virtual hospitals [29] and clinical pharmacy settings [30], but this level of detail is lacking in RACH and GP virtual care delivery settings. One reason for this may be that RACH organizations are yet to define their virtual care processes, procedures, or workflows. Although virtual care frameworks and operational guidance exist within Australian health systems, these are not consistently translated into procedural governance at the RACH level, suggesting challenges related to implementation and dissemination rather than an absence of guidance. At the work system level, the absence of a standardized virtual care consultation process reflects an organizational gap in the provision of formal structures, such as clear procedures and workflows. In the absence of these structures, GPs and RACH staff were required to compensate through increased coordination, decision-making, and cognitive effort to organize and conduct consultations. This reliance on informal, ad hoc processes helps explain the additional workload reported by participants and illustrates how organizational gaps translate into process inefficiencies and increased burden at the individual level. Consequently, existing research has called for standardized virtual care processes to be created within RACH and GP settings, and our findings further support this [31,32].

From a SEIPS perspective, the burden associated with asynchronous email communication reflects an interaction between tasks, organizational workflows, and the external funding environment. While email correspondence emerged as a task performed by GPs, it was experienced as burdensome largely because it is not routinely recognized or remunerated within existing Medicare funding structures. As a result, this form of work becomes effectively “invisible,” contributing to clinician workload and shaping perceptions of virtual care as inefficient or unsustainable. This finding highlights how task-level burdens cannot be fully understood without considering the organizational and policy contexts in which they occur. Recent policy reforms intended to strengthen continuity of care, such as the introduction of voluntary patient registration models, may partially address fragmentation in the general practice relationship with RACH settings. However, where virtual care continues to rely predominantly on fee-for-service billing for discrete clinical encounters, forms of asynchronous coordination such as email triage and communication between RACH staff and GPs are likely to remain undervalued or unpaid. As such, the “invisible” work identified by participants may persist despite policy reform, reinforcing the need for funding models that better align with the realities of virtual care delivery in RACH settings.

Virtual care funding models remain a topic of concern emerging from this study. A scoping review of 23 articles investigated health insurance payment models for virtual care (telehealth) services and found variability across countries [33]. For example, China uses region-specific insurance quotas and reimbursement rates based on hospital level or service price, resulting in varied coverage across provinces [33]. France offers national health insurance that covers 70% of costs, with full coverage for certain groups during the COVID-19 pandemic [33]. In Australia, the universal health system, funded through Medicare, provides low-cost access to public hospital and primary care services [34]. Primary care is largely delivered by private providers in community settings, with patients paying fees offset by standardized Medicare rebates via the MBS [34]. In 2021, virtual care (telehealth) became a permanent feature of Australian primary care with policy rapidly adapted to include changes to MBS items, practice incentive payments, increased scope for GPs to accept Medicare-funded bulk billing payments for telehealth services, and continuity provisions [34,35]. However, our findings reveal that existing funding structures inadequately address the needs of GPs who deliver care to RACHs due to the billing rules, logistical challenges, and a lack of standardized virtual care consultation process. These result in limited collaboration with specialists, challenges in conducting virtual care consults for patients with certain conditions (eg, dementia), clinician frustration, unpaid work, and the propensity to boycott virtual care delivery in RACHs altogether, limiting health care access to residents. With the promise of virtual care being thwarted by poor funding structures and challenges with policy alignment, our findings shed light on how virtual care implementation within this setting in Australia undermines the equitable realization of the SDGs.

The benefits of virtual care delivery in RACHs are well-documented and are consistent with our findings [17]. However, unintended consequences (eg, additional workload, poor uptake, and additional infection control) continue to limit the realization of intended benefits. In our study, virtual care introduced an important system trade-off: while remote consultations reduced GP travel time and supported continuity of care, they simultaneously created additional tasks for RACH nursing staff, such as the new requirement to be present during virtual GP visits and additional infection control tasks related to cleaning and disinfecting shared devices. This illustrates how gains in efficiency for one part of the work system may redistribute workload elsewhere, shaping how virtual care is experienced and sustained in practice. Beyond these trade-offs, our findings also highlight broader systemic constraints. Although virtual care technologies could be more usable and easier to learn, challenges such as lack of system integration and poor network connectivity played a significant role in negatively impacting virtual care delivery, consistent with existing evidence [17,36]. Lack of system integration contributed to duplication of work, particularly when performing virtual care documentation tasks, resulting in additional workload—an unintended consequence for clinicians. Taken together, these findings stress the need for concerted efforts at the macro (government), meso (primary health networks), and micro (RACHs and general practices) levels to tackle work system barriers to ensure the benefits of virtual care can be fully realized, thereby contributing to the earnest achievement of the SDGs.

### Strengths and Limitations

To the best of our knowledge, this is the first study to identify domains of concern to different stakeholder groups, using the SEIPS model to examine work system facilitators and barriers to virtual care delivery between general practices and RACH settings. Our qualitative study was strengthened by the large number of participants (n=32) from multiple RACHs and general practices to provide a complete picture of virtual care delivery between aged care homes and primary care from the perspectives of all relevant stakeholder groups.

Most participants in this study were either RACH staff or primary care providers, whose perspectives were shaped by their roles and responsibilities regarding virtual care delivery. While their insights provided valuable depth regarding work system barriers and enablers, the limited representation from residents and carers may have constrained the breadth of perspectives captured and may further constrain the transferability of findings. It is possible that greater participation from these groups could have yielded additional insights. Future research may benefit from a more balanced representation across stakeholder groups to enrich understanding of virtual care delivery in this setting. To achieve this, future research would benefit from purposive, consumer-led recruitment strategies and methods tailored to the capacities and preferences of older adults and their carers. Also, our findings are based on data collected across one large metropolitan region of New South Wales, Australia, which may limit transferability to other contexts and settings (eg, rural and remote regions). Strengthening consumer representation will be essential for developing virtual care models that are genuinely person-centered and equitable.

### Conclusion

This study generates new evidence to guide how GPs deliver virtual care in RACHs, supporting residents’ access to general practice services and promoting more timely, high-quality care. Our findings have implications for health care organizations seeking to involve GPs in virtual care within RACHs. They also inform investment in virtual care models and technologies, as well as education and training for GPs and RACH staff, alongside policy development by peak bodies and governments. These efforts contribute to SDG 3 (Good Health and Well-Being) by improving residents’ access to timely, high-quality general practice services and to SDG 10 (reduced inequalities) by promoting equitable access to care in aged care settings.

## Supplementary material

10.2196/89019 Multimedia Appendix 1 Interview guide.

10.2196/89019 Multimedia Appendix 2 Participant demographic details.

10.2196/89019 Multimedia Appendix 3 Systems Engineering Initiative for Patient Safety domains, subthemes, and quotes.

10.2196/89019 Checklist 1 COREQ checklist.

## Abbreviations

COREQ: Consolidated Criteria for Reporting Qualitative Research
GP: general practitioner
MBS: Medicare Benefits Schedule
RACH: residential aged care home
SDG: Sustainable Development Goal
SEIPS: Systems Engineering Initiative for Patient Safety
SNHN: Sydney North Health Network
