# Supplementary material for: Virtual Care and Health Care Access: Pragmatic Evaluation of Implementation, Acceptance, and Use in General Practice and Aged Care Homes
Source: J Med Internet Res. 2026 Jun 12;28:e89019. doi: 10.2196/89019 (PMC13263008; doi:10.2196/89019)
Supplement: Multimedia Appendix 1 [file jmir-v28-e89019-s001.docx]

**Multimedia Appendix 1 – Interview Guide**

## Interview questions GPs

**Context**

Date of interview: ________________________________________________________

Time of interview: Beginning: _______________ End: ____________________

Total duration of interview: _________________________

Interviewers (initials): _________________

Participant’s code: _________________________

**Instructions**

Hello, my name is (ENTER NAME), I am part of the research team from the University of Sydney. Thank you for agreeing to be interviewed for this Virtual Care Technologies research project! We are interested in understanding your perceptions of and experiences using Virtual Care technologies. This interview should take around 45 minutes.

Is it okay if I record the interview?

Do you have any questions before we begin?

To start with, can I ask you to fill in this form? (Give pre interview questionnaire if it has not already been completed)

**Interview Questions**

**General impressions of VCT and context of use**

1. What, in your opinion is Virtual Care? and what technologies does virtual care include?
2. How often would you say you use (or have used) VC technology with residents in aged care homes? (prompt: multiple times a week, once a month? not very often at all?)
3. How often would you say you use (or have used) **phone calls** to provide care to residents in aged care homes? (prompt: multiple times a week, once a month? not very often at all?)
4. In thinking back to when you last used a phone call to deliver care to a residential aged care home patient, did it work well?
5. Can you tell me about any challenges you experienced or have experienced when having a telephone consultation? (prompt: organisational, people or technological perspectives)
6. Have you ever used technologies other than phone calls when caring for your residential aged care patients? (if yes go to Q7)
7. Think back to when you last used Virtual Care technologies other than telephone with a residential aged care home patient, did it work well? Can you tell me about any challenges you experienced or have experienced when having a VCT consultation? (prompt: organisational, people or technological perspectives)

**Impact on overall work system**

1. Thinking about the last time you had a virtual care consultation; can you walk me through the process? What does it usually entail? (prompt: how do you know that your next patient will be a virtual care style consultation? Would you mind showing me/describing to me how you would go about logging on to your system now and showing me what you would do?)
2. Is there a different approach based on the technology being used? (follow up: how are they different?)
3. What features or functions might improve the current virtual care technology you use?
4. Do you think there are any benefits in using VC technology for RACH patients? If so, what do you think they are? (follow up: if not why?)
5. In what instances do you think that virtual care technology can’t or shouldn’t be used for residents?

**Use of VC implementation guidance**

1. Could you tell me about any implementation guidance you are aware of to help you undertake virtual care consultations with RACH patients specifically? Which guidance materials have you used if any? (e.g. PHN, RACGP guidelines or other resources, or websites)
2. What about other guidance materials for Virtual Care or telehealth consultations **in general**? Are you familiar with any resources and have you used any in the past? If so, which ones?
3. Have you ever used the Health Teams platform? If yes – go to Q 16, if no - go to Q 18

**Use of Health Teams and VC implementation guidance**

1. In thinking about the Health Teams implementation (i.e. roll-out) to your practice, can you describe how it was rolled out and what worked well or didn’t work well? (prompt: training, resources, etc – follow up: who gave training, what resources were given)
2. Can you think of any improvements needed to the way Health Teams was rolled out?
3. Any other comments?

**Recruitment**

1. Do you know any other GPs or PMs who may be interested in participating in our study?
2. Do you know any nurses/residents/carers who may be interested in participating in our study?

Thank you for your time today, it’s really appreciated. An e-gift card will be sent to your email address.

## Interview questions RACH RN

**Context**

Date of interview: ________________________________________________________

Time of interview: Beginning: _______________ End: ____________________

Total duration of interview: _________________________

Interviewers (initials): _________________

Participant’s code: _________________________

**Instructions**

Hello, my name is (ENTER NAME), I am part of the research team from the University of Sydney. Thank you for agreeing to be interviewed for this Virtual Care Technologies research project! We are interested in understanding your perceptions of and experiences using Virtual Care technologies. This interview should take around 45 minutes.

Is it okay if record the interview?

Do you have any questions before we begin?

To start with, can I ask you to fill in this form? (Give pre interview form if it has not already been completed)

**Interview Questions**

**General impressions of VCT and context of use**

1. What, in your opinion is Virtual Care? and what technologies does virtual care include?
2. How often would you say you use (or have used) VC technology with residents in aged care homes? (prompt: multiple times a week, once a month? not very often at all?)
3. How often would you say you use (or have used) **phone calls** to provide care to residents in aged care homes? (prompt: multiple times a week, once a month? not very often at all?)
4. In thinking back to when you last used a phone call to deliver care to a resident, did it work well?
5. Can you tell me about any challenges you experienced or have experienced when having a telephone consultation? (prompt: organisational, people or technological perspectives)
6. Have you ever used technologies other than phone calls when caring for your residents? (if yes go to Q7)
7. Think back to when you last used Virtual Care technologies other than telephone with a resident, did it work well? Can you tell me about any challenges you experienced or have experienced when having a VCT consultation? (prompt: organisational, people or technological perspectives)

**Impact on overall work system**

1. Thinking about the last time you had a virtual care consultation; can you walk me through the process? What does it usually entail? (prompt: how do you know that your next patient will be a virtual care style consultation? Would you mind showing me/describing to me how you would go about logging on to your system now and showing me what you would do?)
2. Is there a different approach based on the technology being used? (follow up: how are they different?)
3. What features or functions might improve the current virtual care technology you use?
4. Do you think there are any benefits in using VC technology for RACH residents? If so, what do you think they are? (follow up: if not why?)
5. In what instances do you think that virtual care technology can’t or shouldn’t be used for residents?

**Use of VC implementation guidance**

1. Could you tell me about any implementation guidance you are aware of to help you undertake virtual care consultations with RACH residents specifically? Which guidance materials have you used if any? (e.g. PHN, other resources or websites)
2. What about other guidance materials for Virtual Care or telehealth consultations **in general**? Are you familiar with any resources and have you used any in the past? If so, which ones?
3. Have you ever used the Health Teams platform? If yes – go to Q 16, if no - go to Q 18

**Use of Health Teams and VC implementation guidance**

1. In thinking about the Health Teams implementation, can you describe how it was rolled out and what worked well or didn’t work well? (prompt: training, resources, etc – follow up: who gave training, what resources were given)
2. Can you think of any improvements needed to the way Health Teams was rolled out?
3. Any other comments?

**Recruitment**

1. Do you know any residents/carers who may be interested?

Thank you for your time today, it’s really appreciated. (Give gift card.)

## Interview questions Practice Managers

**Context**

Date of interview: ________________________________________________________

Time of interview: Beginning: _______________ End: ____________________

Total duration of interview: _________________________

Interviewers (initials): _________________

Participant’s code: _________________________

**Instructions**

Hello, my name is (ENTER NAME), I am part of the research team from the University of Sydney. Thank you for agreeing to be interviewed for this Virtual Care Technologies research project! We are interested in understanding your perceptions of and experiences using Virtual Care technologies. This interview should take around 45 minutes.

Is it okay if I audio-record the interview?

Do you have any questions before we begin?

To start with, can I ask you to fill in this form? (Give pre interview questionnaire if not completed prior)

**Interview Questions**

**General impressions of VCT and context of use**

1. What, in your opinion is Virtual Care? and what do you think virtual care technologies include?
2. What is your role in using or facilitating the use of virtual care technologies for residents in aged care homes who are cared for by members of your practice team?
3. If used, what kind of telehealth technologies are used in this practice for Virtual Care with residents from aged care homes? (prompt: telehealth, facetime, teams, zoom, photographs sent using a messaging service).

**Impact on overall work system**

1. Thinking about the last time you supported or arranged a virtual care consultation to one of the GPs here, can you walk me through the process? What does it usually entail? Would you mind describing to me/showing me how you would go about logging on to your system now and showing me what you would do?
2. Is there a different approach to the way you arrange a virtual care consultation based on the technology being used? (follow up: how are they different?
3. Is there a different approach to the way you arrange a virtual care consultation based on the GP’s preference? If so, what do you think influences this?
4. In thinking about the RACH appointments you organise for the GPs in the practice via virtual care, can you tell me about any challenges you have experienced? (prompt: organisational, people or technological perspectives)
5. How do you think this may be remedied or made easier?
6. Have you or your team members ever used Health Teams platform? If yes – go to Q 10, if no - go to Q 12

**Use of Health Teams and other VC implementation guidance**

1. In thinking about the Health Teams implementation to the practice, can you describe how it was rolled out, what worked out and what didn’t work well? (prompt: who gave training, what resources were given)
2. Can you think of any improvements needed to the way Health Teams was rolled out?
3. Do you or others in your practice team that you are aware of use any specific guidelines or other support information or materials to guide the way your practice delivers virtual care or telehealth consultations?
4. Any other comments?

Thank you for your time today, it’s really appreciated. (Give gift card.)

## INTERVIEW QUESTIONS RACH RESIDENT

**Context**

Date of interview: ________________________________________________________

Time of interview: Beginning: _______________ End: ____________________

Total duration of interview: _________________________

Interviewers (initials): _________________

Participant’s code: _________________________

**Instructions**

Hello, my name is (ENTER NAME), I am a research fellow from the University of Sydney. Thank you for being willing to be interviewed for this Virtual Care Technologies research project! We are interested in understanding your perceptions of and experiences using VCT. The interview should take around 30 minutes.

Is it okay if I audio-record the interview?

Do you have any questions before we begin?

To start with, can I ask you to fill in this form? (Give pre interview questionnaire if not completed prior)

**Interview questions**

**General impressions of VCT and context of use**

1. What do you understand the term virtual care technology to mean?

*If the participant doesn’t understand the term virtual care technology, the interviewer will say “When we talk about virtual care technology, we mean care that you receive that is not in-person with your GP or other health care provider. For example this might be using a phone or a video call.”*

1. If you think about your experiences with virtual care, do you feel it has worked well or that there have been any issues?
2. Apart from your GP does anyone else who provides your health care use virtual care technologies? (prompt: for example, facetime, teams, zoom, a photo taken and sent using a text or other message to your doctor?) and if so who?
3. (If the carer is part of the interview) As a carer, have you participated in any virtual care consultations with (RESIDENTS NAME)? (IF YES) If you think about your experiences with virtual care, do you feel it has worked well or that there have been any issues?

**Resources and implementation guidance**

1. Has anyone spoken to you or given you any written advice about the way virtual care technology or telehealth could be used in residential aged care homes in the past? If yes, what was that information and did you find it useful?
2. Would you like to tell me anything else about your experiences with virtual care?

Thank you for your time today, it’s really appreciated. (Give gift card.)

## INTERVIEW QUESTIONS RACH CARER

**Context**

Date of interview: ________________________________________________________

Time of interview: Beginning: _______________ End: ____________________

Total duration of interview: _________________________

Interviewers (initials): _________________

Participant’s code: _________________________

**Instructions**

Hello, my name is (ENTER NAME), I am a research fellow from the University of Sydney. Thank you for being willing to be interviewed for this Virtual Care Technologies research project! We are interested in understanding your perceptions of and experiences using VCT. The interview should take around 30 minutes.

Is it okay if I audio-record the interview?

Do you have any questions before we begin?

To start with, can I ask you to fill in this form? (Give pre interview questionnaire if not completed prior)

**Interview questions**

**General impressions of VCT and context of use**

1. What do you understand the term virtual care technology to mean?

*If the participant doesn’t understand the term virtual care technology, the interviewer will say “When we talk about virtual care technology, we mean care that you receive that is not in-person with your GP or other health care provider. For example this might be using a phone or a video call.”*

1. Have you participating in any virtual care consultations with (RESIDENT’S NAME)? (IF YES). If you think about your experiences with virtual care, do you feel it has worked well or has there been any issues?
2. When (RESIDENT’S NAME) has participated in a virtual care consultation do you feel it has worked well for them or has there been any issues?
3. Apart from your GP does anyone else who provides (RESIDENT’S NAME) health care use virtual care technologies? (prompt: for example, facetime, teams, zoom, a photo taken and sent using a text or other message to your doctor?) and if so who?

**Resources and implementation guidance**

1. Has anyone spoken to you or given you any written advice about the way virtual care technology or telehealth could be used in residential aged care homes in the past? If yes, what was that information and did you find it useful?
2. Would you like to tell me anything else about your experiences with virtual care?

Thank you for your time today, it’s really appreciated. (Give gift card.)
