# Supplementary material for: Virtual Care and Health Care Access: Pragmatic Evaluation of Implementation, Acceptance, and Use in General Practice and Aged Care Homes
Source: J Med Internet Res. 2026 Jun 12;28:e89019. doi: 10.2196/89019 (PMC13263008; doi:10.2196/89019)
Supplement: Multimedia Appendix 2 [file jmir-v28-e89019-s002.docx]

**Multimedia Appendix 2 -** Additional participant demographics

*General practitioners*

GPs reported a wide range of allied healthcare providers working at their practice including physiotherapists (n=3), podiatrists (n=2), dietitians (n=2), gastroenterologist (n=1), urologist (n=1), obstetrician (n=1), speech therapist (n=1), massage therapist (n=1) and nurse practitioner (n=1). Three other GP participants reported no allied healthcare providers.

**Table 1**. GP participants virtual care usage

| Technology used in practice, n (%)  Phone  Phone and messaging platform  Phone, video telehealth platform, messaging platform  Phone and video telehealth platform  Technology used for virtual care with residents, n (%)    Phone  Phone and messaging platform  Phone, video telehealth platform, messaging platform  Video telehealth platform and messaging platform  None  Approximate proportion of phone vs video telehealth consultations, n (%)  < 50% phone  > 50% phone  100% video  How often video telehealth technologies used in RACH, n (%)  Not in use  Not often at all  Multiple times a week  Unsure | Value  5 (45)  1 (9)  4 (36)  1 (9)  3 (27)  1 (9)  3 (27)  1 (9)  2 (18)  7 (63)  2 (18)  1 (9)  4 (36)  4 (36)  2 (18)  1 (9) |
| --- | --- |

*RACH staff and practice managers*

Most registered nurses (RNs) work at one RACH except for three RN participants who also support several others. Most RNs work 5 days per week (n=8). One RN works more than 6 days per week and 2 other RN participants work 4 days per week. All PM participants have a nurse working in their practice. Most PM participants have their practice nurse involved in the care of RACH patients (n=2).
