# Supplementary material for: Virtual Care and Health Care Access: Pragmatic Evaluation of Implementation, Acceptance, and Use in General Practice and Aged Care Homes
Source: J Med Internet Res. 2026 Jun 12;28:e89019. doi: 10.2196/89019 (PMC13263008; doi:10.2196/89019)
Supplement: Multimedia Appendix 3 [file jmir-v28-e89019-s003.docx]

**Multimedia Appendix 3.** SEIPS domains, subthemes, and quotes

| **SEIPS elements** | **Category** | **Sub-themes** | **Quotes** |
| --- | --- | --- | --- |
| **Work system elements** | | | |
| People | Description of how providers determined virtual care suitability for residents | Complexity of care needs | “*end of life care is something that I think you need, that personal touch is very important*” (P2, RN).  “*I really can't think of a reason, and I don't think palliative care is one, if anything, are you gonna leave somebody to suffer while you're waiting for a doctor to come in person? You're not gonna leave somebody for any reason when there's an alternative that will potentially work just as well*.” (P6, RN). |
|  | Dual traits | Digital literacy | Enabler: “*she's 100 years old … she was able to just quickly put me on FaceTime so I could see her legs and see if they're getting better*” (P3, GP).  Barrier: “*I don't get a good view of the patient … the physical call itself, is sometimes a bit useless because the person doing it is not maybe very well educated in telehealth*” (P17, GP) |
|  |  | Familiarity | Enabler: “*So over the phone, I felt the same relationship from all the years that have been going to [the GP]…Yeah, it felt like it was in person. So my thinking has distinguished between like a well-known person, very common, very good relationship, and she was always straight about anything*” (P31, resident).  Barrier: “*But when I didn't know the person and didn't have a relationship, it was always more difficult, and even more so when other people were between me and the other person…*[if familiar] *I didn't feel like I lost anything in not being with the person”* (P31, resident). |
|  |  | Cognitive functioning of residents | Barrier: “*it could be bit challenging, and sometimes residents with dementia here, they may not understand the process, and they are more resistant and hesitated*” (P12, RN). |
|  | Barriers | Communication challenges | “*it wasn't the doctor's fault, it was the way the staff members were…And English isn't their first language*” (P31, resident). |
|  |  | The potential to exclude residents from conversation | “*He does everything I want on the phone and not direct with me, just the nurse. I tell the nurse, yeah, and she goes I will talk to him. She comes back and interprets whatever he says. I've never seen him. I don't know if he exists*” (P29, resident)  “*Well, the whole problem is, in when they go into aged care, you haven't got that direct contact with the doctor. The RN usually takes over that… I would rather talk to the GP about the situation, or my husband should talk to the GP about his situation... But that's what happens when you go into aged care. You don't have that autonomy*” (P30, carer). |
|  |  | Limited knowledge level of RACH staff | “*If you have your care staff sitting there, there's different levels of knowledge of the care staff, and sometimes they may not understand what the doctor is asking them, and therefore sometimes confusion has developed*” (P2, RN)  “*If nurses can't do assessment properly … sometimes nurse’s assistant might mislead and misguide* [the doctors]” (P12, RN).  “*they could technically get an enrolled nurse to facilitate and do it [virtual care call], or one of the care workers, but then you're still going to have the issue of them passing on the information appropriately…it depends who you've got…even when you pass on the information one day, three days later, none of the treating team are aware of the information*” (P5, GP). |
|  |  | Resistance to using new technology | “*for the registered nurses …they are used to the old ways. They are not very open to the new technology*” (P9, RN)  “*it's so frustrating to see that like us as GP…we're really resistant to change*” (P4, GP).  “*a lot of the older GPs they really have a bad sentiment towards telehealth… They hate it. They think it's not proper medicine… So there is bad optics around it also, and they may have had bad experiences around it*” (P17, GP). |
|  |  | GP preference and willingness | “*I met some doctors who don't even want to use any iCare or electronic documentation, everything they wrote it down on the progress notes is hand-written. Yeah, and he's still practicing, in my last nursing home*” (P1, RN).  “*GPs can't be bothered to learn new things. So it's not that they are not promoting it, but I think part of it, just that GPs, they just want to stay the same. …We don't want to make changes, especially if you are busy*” (P4, GP). |
| Tasks | Description of tasks that trigger virtual care consultation requests to GPs | Tasks that trigger either phone or video consultation requests | “*Most of the scenarios that happen is like deteriorating wound, wound care … or skin problem, or bruising, or anything that is not life-threatening*” (P1, RN) |
|  | Pre-consultation tasks | Appointment booking | “*if the matter is urgent, that has to be done in the next half an hour. One of the first things we do is that we'll just call the GP. Just make a call, and if the GP is not picking up the phone, then we send them a text message, and most of the time they will reply, or they will call us back*” (P1, RN).  “*it's different with different GPs, as I mentioned you earlier, one of the GPs, he doesn't accept phone call apart from the time he has given. So we don't call him out of that time, if there is emergency, either a BRACE team or a hospital, but some other GP, they say, okay, you can call us anytime during the day*” (P12_RN). |
|  |  | Vital signs data collection | “*we ask the receptionist to ask the doctor if they want us to do any observations, blood pressures, all those sort of things prior to the ring so we've got that information available*” (P2, RN).  “*you'd have to ask for it, and it's not going to get done before, unless it was like, someone who was sick and like, unless you've asked for it prior to the appointment they won't. They're all too busy. So they should, but they don't*” (P3, GP). |
|  |  | Review of patient records | “*it depends on the consultation. But like say, as an example, there's a issue with someone living with dementia with ptsd, and they want me to review their behaviors, so I would, before doing the consultation, log on to their CMS. Have a look at all the RN notes. Okay, it's very important for me. Have a look at sleep charts, have a look at bowel charts, have a look at pain charts, right? What are all the triggers of these behaviours? So I've got context to what then I'm doing, because the consultation itself might not be that important*” (P17, GP). |
|  | Post-consultation tasks | Documentation | “*if it's basically weekend, or if it's basically, I am out of Sydney, so I don't have access to my own PC at home to get access to the remote access to the Best Practice. Then I will get nurse to write on my behalf. Then basically, by the time that I get to the telehealth or to the Sydney I have access either to my remote control at home or at the practice, I'll do my notes as well*.” (P18, GP)  “*But all the telehealth that I’ve done here so far, I can tell you, 100% I was the only one that documented, it was not the doctor*” (P16, RN).  “*the doctor will say, okay, we'll do this, we'll do that before our meeting finishes, I will say, are you sending us the reports by email? Or we'll just do this? And sometimes they will say, okay, we will send you an email of what the plans are. It depends*” (P24, RN). |
|  | Barriers to task completion | Emails from nurses in RACHs pass along the burden of responsibility | “*Email has actually made things very difficult, because people think that by sending an email, their job is done. So you know, like, if you're in care, a nurse might say, resident has a rash, and I got this email ... So now it's up to me to make that appointment… So then often you reply to the email saying, fix an appointment, call me or I'll do a telehealth at two o'clock... And often they won't call, or they may call, and if you ring back, then again, I don't know the RN who has called me. I may not read the email straight away. They might have finished their shift*” (P13, GP).  “*We tend to discourage emails because it's hard to know when they'll be acted on, and because they have the practice email, not my personal email, and things like that. So emails are fine for advising me of something like where an action doesn't need to be taken*” (P15, GP).  “*care management should be liable at the facilities for that clinical handover, right? Like if they requesting virtual care, then there should be someone responsible for ensuring that there's clinical continuity, and that has to come from their side*” (P17, GP).  “*We prefer email because at least it's documented*” (P26, RN). |
|  |  | Interruptions | “*he doesn't ever turn his things off properly. Even when we're in the room, there'll be people you know in the room or you can hear the click of his phone that he's got in front of him… Apparently they [other clinicians] aren't allowed to do some things without his authorisation. So rather than hold them up all the time, they come in and interrupt you as you are talking…And he tended to be combining sometimes, dealing with me and dealing with something coming over the computer*” (P31, resident). |
|  |  | Duplication of work | “*When you've got to repeat what you’ve done potentially three or four times, you know, whether you're a PDF document or you cut and paste, it's a real issue*” (P6, RN). |
|  |  | Inefficiencies | “[documentation] *is a big pain point for a lot of clinicians, it takes some time to upload their notes*” (P17, GP). |
|  | Enablers of virtual care consultation task | Ability to conduct a visual assessment | “*I think the biggest thing is always just, it's just being able to see the patient. I think that's the main thing. To be able to see them and talk to them and see what they're doing is a massive deal*” (P3, GP) |
|  |  | Ability to collect and share vital signs data | “*I think it's just having all of the information at hand, and I think that's what HealthTeams is trying to do, which I appreciate, right? Like, so, as with any consultation, you want context of what you're doing. So you know, if someone, if you're reviewing someone for increasing shortness of breath, you want to make sure that you have access to their recent weights, and you know what I mean, and looking at their charts and having all of that information in an accessible place*” (P17, GP). |
| Tools and technology | Description of technologies and tools supporting virtual care delivery | Tool – paper-based medication chart | “*we* [are] *still using the paper-based medication chart. So what we do is we scan the chart in PDF format, and then we email doctor, the doctor changes it, [and] sends the chart back to us*” (P1, RN). |
|  | Dual traits | Screen size | Barrier: “*But it's only a mobile phone. It's not big enough for the residents to see. They need a visual screen where they can see their GP or see the practice, and then they feel comfortable*.” (P2, RN)  Enabler: “*So, like, a bigger screen, or some sort of, like, you know, efficient manner of doing it that would probably help*” (P15, GP) |
|  | Barriers | Lack of system integration | “*the huge amount of fragmentation* [that exists]*, and the fact that providers are just not going to be able to login to all these different systems and waste half an hour to upload one single consultation*” (P17, GP).  “*we essentially have three different software. I've got Best Practice for me, and I've got best med for the medication charts. And then they've got their software…and I don't have access to it… And so we have to, then at the end of my consult, print off my notes to send to them, or I cut and paste them and email them to the nursing home, because it doesn't liaise with Best Practice*” (P3, GP).  “*each nursing home has different software in place that doesn't link up to their GP software. There's no national software that they use. They have a national medication chart now…they give a GP a login, and you only get access to the nursing home that you regularly visit. So if you're then trying to cover for another GP in your practice who visits another nursing home, you don't even have access to those residents’ medication charts. And now, if a patient goes to hospital and then they get their medication changed in hospital, that doesn't get updated on the patient's medication chart, because the hospital doesn't have access to their national medication chart… the whole thing's a nightmare*” (P5, GP).  “*there's no integration with the clinical software that I use currently in the residential aged care homes that I'm involved with…which makes it very difficult*” (P6, RN). |
|  |  | Poor usability | “*not very user friendly*” (P26, RN),  “*not easy to learn*” (P17, GP),  “*clunky, … inefficient, because you have to talk through it with them*” (P20, GP). “*forget how to navigate it*” (P26, RN)  “*But sometimes, if we use a tablet, it's about whether we all can remember how to set it up and get it ready*” (P7, RN).  “*So you need to set the audio and video setting very carefully. Otherwise, once you muck up the first time you're not going to, you have to use the other phone pretty much. If they are unable to hear, or they see the picture, or see the resident, it's not going to do the virtual care. So there's some kind of, I think it's like a browser setting or firewall setting that we need to disable it. So once you get through that step, you'll be fine, yeah, but sometimes the nurses will accidentally click something that disable the voice or disable the video, and then the whole pop-up*” (P1, RN). |
|  |  | Poor network connectivity | “*you're always constrained by things like reception, and really good network coverage in residential aged care, there's always a black spot, like there is always a spot that may not have the best coverage*” (P27, RN).  “*good internet, so enough bandwidth to facilitate a good, clear call, you know, without it being choppy. And so that's number one. And then also, you know, that affects audio. It affects the clarity of the video. You know, if I'm doing a telehealth review of the wound, if I get really grainy, terrible video, it's of no value… So sometimes, in those cases, I will then get the RN to send me a high-resolution photo via text, okay, just to back up that kind of review of the wound*” (P17, GP). |
|  |  | Inability to conduct full assessment | “*the challenge is that I cannot see anything. It's just like verbal and sometimes, if I'm worried about something and [to] make sure nothing is wrong, then I still have to go in*” (P11, GP) |
|  |  | Difficulties accessing clinical information systems | “*a lot of facilities require you logging in via VPN. ... Every single provider uses a different VPN, and it's a real - it's a nightmare. Like I said, like, I am fine with it, but I know most, like probably 90% of other clinicians, wouldn't be able to so then how do we deliver the clinical handover without doing that*?” (P17, GP) |
|  |  | Difficulties accessing IT support | “*getting the IT to set it up can be a bit of a roadblock for me. Because each computer would need to be set up, and then IT is not on site. So getting them to come in is another issue, and getting it all set up. Because, you've always got doctors in the room, so you can't get in at a particular time, so IT would have to keep coming back. And so that's why we just stuck with phones, just easier to do that.*” (P8, practice manager) |
|  |  | Technology glitches | “*it's face to face more so because of the difficulties faced with this technology, you know, the glitches and … It's too hard sometimes*.” (P6, RN) |
|  | Enablers | Third-party video software | “*WhatsApp and FaceTime is actually much easier and quicker, because you can just tell the nurse, okay, WhatsApp me, and you know, it happens within two minutes. You're not faffing around with links and codes and all that. You know, they don't have to go into a browser and click on a link and all that kind of things. So that works quite quickly*” (P13, GP).  “*with FaceTime, I think nursing homes have no problem*” (P4, GP). |
|  |  | Remote access to clinical information systems | “*the doctor has got access to two software, mainly the main software, which is a nursing home software, and we don't see it here, that's within her tablets that she uses, or iPad, and that software is centralised, that's from the nursing home, so that our doctor uses that to document…which she can also remotely access from her home or in the practice*” (P21, practice manager).  “*use BestMed. And … GP has access to BestMed from anywhere. … We use iCare and all our GPs they have a record, they have access to iCare*” (P14, RN).  “*so I could, wherever I am, actually look at the notes that if they've done something, I can see. Also the prescription, the medication chart was also online as well, so it's easy when I need to prescribe something*” (P4, GP). |
| Organisation | Dual traits | Awareness | Enabler: “[Let residents know] *that these [virtual care] options are professional and that they're available and they're tested and they work*” (P2, RN)  Barrier: “*because a lot of our families just say, oh, ring an ambulance…When some people don't need to have gone to an emergency department*” (P2, RN). |
|  |  | Availability of portable smart devices | Enabler: “*If I was performing the consult, I would do it for my phone… because I find it easier to manoeuvre. I'm needing to move the camera and things like that as opposed to a computer*” (P27, RN).  Barriers: “*We don't have access to iPads…But it's only a mobile phone. It's not big enough for the residents to see. They need a visual screen where they can see their GP … so definitely that is a resource issue at the moment*” (P2, RN).  “*at the moment, if we do have to have a telehealth, it needs to be done by our own private phone, which is not really ideal. We don't have anything in place at this stage… [we need RACH organisations to] buy a device for the nurses, because we need [it]”* (P16, RN).  “*in reality, most of these facilities, most of the telehealth is done by an RN on her phone*” (P17, GP), |
|  |  | Training | Barriers: “*Not so much into training on how to use video call. They come in to provide the support, like how to do our initial consultation, what information the nurses will need to cover. But not so much on teaching, step by step. Oh, you need to log into the website. I need to disable ABC. Not so much…I got stuck once, and my other RN and they got stuck too. And then at the end, we were able to dial in, but we can't hear anything. They don't see anything because of the firewall thing. Not so much on how to use*” (P1, RN).  “*you need to also train staff to sit there with the resident…because a lot of our care staff so we have registered nurses here during the day, but we have care staff over the night. We don't have registered nurses overnight here. So therefore, if there's an issue, some of our staff only have six weeks training*” (P2, RN).  “*And I was thinking why don't you turn it around and come closer to me? But they were far away from the bed, and so that wasn't at all satisfactory*” (P31, resident). |
|  |  | Management of privacy | Barriers: “*We don't have a separate room. I went to resident's room*” (P14, RN).  “*even when we're in the room, there'll be people you know in the room*” (P31, resident)  “*if you apply the rules about privacy and all that you're supposed to ask them, you're supposed to ask them [whether] they're happy to have the nurse there. So in an ideal world, the nurse would leave the video screen with them and walk out, and that's just never going to work. I can't imagine speaking to a patient with dementia who's holding a laptop or something and asking them, you know, how do you feel? Can you show me your leg? Or does this hurt or that hurt? That's never going to work*” (P13, GP).  “*FaceTime would be more effective, but I don't use that because it gives my phone number to the person, which I don't want to do*” (P20, GP).  “*using [my] private phone, which I totally disagree, because I don't want to have anything to do clinically, and I don't want anyone to know my number unless it's really necessary. So officially, I was put in a position that I had to give my personal number, completely against my wishes*” (P16, RN). |
|  | Barriers | Logistical challenges | “*most of the time we're trying to get this to be right, because you can't claim Medicare, and therefore you can't be paid unless you have the client there. So what often happens is you try and ring the nurse. The nurse tries to find the client, then the client and the nurse have to call you back, by which time you’ve already wasted your 15 minute consulting and you're now running late … and that's if you find the nurse at the time of the consult…and if you find the patient at the time of the consult, so it's a disaster. It's been really hard*” (P3, GP).  “*there's no planned phone calls and it's not as structured as we hope it would be. So for a phone call, obviously we want to use ISBAR when we're talking to the GPs, but sometimes they would say that okay, but I'm with a client right now, and I understand what you need from me, but can we do a follow up on this? So then we end up following up the next day and then the next day after to get the point across*” (P26, RN). |
|  |  | Organisational policies and procedures | “*that an issue is the company policies on various issues that happens or incidents that happen. We have policies that mandate that we have to transfer to people to hospital to be investigated…so that's something to think [about] and each organisation has different policies and procedures around transfer to hospital*” (P2, RN).  “*the legislation hasn't caught up*” (P13, GP). |
|  |  | Limited working hours | “*sometimes they will call outside work hours…So back when I was working in the rural place, I was picking up after hours, but then in Sydney, I wasn't able to pick up after hours*.” (P11, GP) |
|  |  | Staff turnover | “*they have a lot of changeover on agency staff which would be trying hard to sort of catch up on previous information and might not quite know what's going on*.” (P5, GP)  “*Nurses keep changing. So the consistency is difficult, and Facility A might work very well, and then suddenly somebody comes and messes things up, or management changes…so there is staff turnover. How many are you going to train up?*” (P13, GP).  “*it probably is a matter of just the revolving door of staff in a lot of aged care, so a lot of agency staffing…It's very rare to see permanent staffing*.” (P17, GP). |
|  | Enablers | Availability of registered nurses during the virtual consult | “*Thankfully, the RN have been quite good, so they'll often check things for me, like, if there's fetal edema, where does it go up to? If a patient's got a wound or something, they'll send me a photo while they're there… and you obviously don't know what you're missing, but I haven't found it too bad*” (P15, GP).  “*So if a registered nurse is sitting there with the resident, then the phone call went really well*” (P2, RN). |
| Environment | Internal environment barriers | Proximity to care delivery facilities, | “*No videos, no, not at all. Because we have the nursing home next to our GP clinic. So in case we need to see them, we just pop in consultation that way… we are literally next door*” (P21, Practice manager).  “*The nursing home that I was with was only one suburb away from my surgery, so it was easier to get there too*” (P4, GP). |
|  |  | Noise | “*the voice* [was] *not clear due to the background noise*” (P10, practice manager). |
|  | External environment barriers | Challenges with funding model | “*And the problem is that they've made it so immensely difficult … to access those incentive payments that they've kind of shot themselves in the foot… not only is it hard to sign people up, to actually get it, you have to be working in an accredited practice. And then, on top of all of that, … are these sort of clinical benchmarks. You have to see a patient twice within three months and all throughout the year. And if you don't hit those milestones, you don't get the payment. So it's really complicated, and it's really confusing why they did it that way*” (P17, GP).  “*most of the time we're trying to get this to be right, because you can't claim Medicare, and therefore you can't be paid unless you have the client there. So what often happens is you try and ring the nurse. The nurse tries to find the client, then the client and the nurse have to call you back, by which time you’ve already wasted your 15 minute consulting and you're now running late, and that's just getting started, and that's if you find the nurse at the time of the consult, so you know, and if you find the patient at the time of the consult, so it's a disaster*” (P3, GP)  “*it's half the pay of what you usually get for anything else*” (P5, GP),  “*So there's still a lot of unpaid work being done at my nursing home…which is really like frustrating*” (P3, GP). |
| **Process** | | | |
| Virtual care consultation process | Current process | No defined process | “*We don't have a process as such…We don't have that. I have never even looked into if there is a policy of the video call, what exactly you're supposed to do*” (P16, RN). |
|  |  | Varying approaches | “*I generally call RN and then they will go to the patient room. And often, if they've got decent hearing, and there's not to much of an issue with the connection and things, we tend to just go on to speaker phone so everyone can kind of hear what's happening. And for some residents, works fine. Or if the issue itself is really quick, like something needs to be charted, or whatever securely, just from a Medicare point of view, to have the consultation, then yeah, that that works well as well. But occasionally, if the patient is hard of hearing, or, for example, they're actually someone with dementia who's not really able to contribute to the consultation, then it maybe they're there, but I'm just talking to the RN*” (P15, RN). |
|  |  | Informal consultations | “*So the nurses at the nursing home might send an update on a patient's clinical condition and ask me what I'm going to do about it, or they must send me a photo of their wound that needs reviewing*” (P19, GP).  “*I don't charge it. I don't put that through Medicare, because it's just too complicated and too annoying to go through the whole process of getting consent and the rest...It's a non-formal thing to sort out the patient for that night or for that time till I go and see the patient the day after or two days later*” (P25, GP). |
| **Outcomes** | | | |
| Patients | Benefits to patients | Improved access to care | “*they* [residents] *get access to a lot more clinicians than just GP that is in their local GP centre, that's a huge benefit, because otherwise, obviously they would have only one GP who's very busy…and cannot come in … They can actually have more care provided by doctors*.” (P20, GP). |
|  |  | Minimises unnecessary hospital visits | “*I've used it [virtual care] very much successfully to prevent hospital admissions in an acute escalation, sort of pathway*” (P27, RN) |
|  |  | Minimises disruptions to residents’ familiar environment | “*for a lot of residents, they don't want to get transferred to hospital because it's an unfamiliar environment. They will get high risk of delirium… so if it's critical and GP is not available in person, …I think that's where the virtual care will kick in*” (P9, RN). |
|  |  | Positive impact on resident’s quality of life | “*if the resident will see the doctor on the video, they feel more like they feel more comfortable, like they feel safe, oh, that they can see the doctor. You know how residents are if, if they see their doctor, they're happy. When the doctor is not there, they feel like this and like that*.” (P24, RN) |
|  |  | Providing continuity of care | “*if I had access to a system that I could see the patient, I could do my consult from where I am … while I'm away to do the work and continue the patient care…So it would add to the continuity of care, in a way*.” (P25, GP) |
|  |  | Providing a safer alternative when compared to audio-only telehealth calls | “*I feel as though a video is more effective…Like I said to you, you miss out on some core things, that's why I kind of advocate more for a video call, because I feel like you can really miss out on some really core information, and the video is such a simple option that we can do*.” (P27, RN)  “*if I had access to a system that I could see the patient, I could do my consult from where I am.... But phone calls, purely a phone call, is not safe enough. It doesn't feel safe enough to do that*. (P25, GP) |
|  | Unintended consequence to patients | Loss of doctor-patient relationship | “*There's something that happens when you're in a room with someone…it's just a human-to-human interaction. Yeah, there's something that it's just lost … you know that patient-doctor relationship, there is something about…meeting people, safe in their hand*” (P17, GP). |
| Clinicians | Dual trait | Efficiency | “*like myself, most GPs are working somewhere else, it saves the travel time, allowing us to do more… you can see more patients at the nursing home*” (P15, GP).  “*So if I say I'm going to do telehealth at three o'clock, I'm just sitting around, often they won't ring me at the time. Or when they ring me…they're ringing on the phone. I said, no, I want video. And they'll say, Okay, I'll go to the room and ring you back. It's very time consuming*” (P13, GP). |
|  | Benefits to clinicians | Access to visual cues | “*from [a] clinical side of things I'm always worried about when a patient is unwell looking, so a phone call doesn't give me that information…you need to see them to see how unwell they are, to decide if they need to go to hospital or if it can wait for another 24 hours. So that visualisation of patients makes a big difference. And I guess that's when I use the video call, video consult*” (P25, GP).  “*I feel like with a video call, once you can see them, you can also get a lot more information out of it, and you can do some very minor examinations over a video conference with … the assistance of a nurse*” (P27, RN). |
|  | Unintended consequence to clinicians | Additional workload | “*And there's other things... they've got to do with assessment and maintaining the aged care quality standards … that keeps them time poor … because they're trying to do this paperwork. And, I think those reasons are the reason they see this as [extra workload] …the impact of this on our already busy life, or work life, does put a strain on them…and the workload. The workload is huge*” (P6, RN).  “*The only thing we found is, even for the teleconference, we found a bit of difficulty with time management, because we have 61 residents in our facility. We have two RNs, so each will get 30 patients and if I need to stand in front of the computer to deal with whatever situation this resident have, what about the rest of the residents*” (P9, RN). |
|  |  | Inability to conduct physical examinations | “*the inability to physically examine, which is far more important if you have someone that's not able to communicate with you*” (P5, GP). |
| Health service | Dual trait | Infection control | “*I will say, infection control, because my own phone was in an area where after all this I had to disinfect my phone ... And I think really, we shouldn't mix our own personal phone with facility problems that they might encounter. So we should have one designated device inside every nursing home*” (P16, RN). |
|  | Unintended consequence to the health service | Poor uptake | “*the idea of someone being able to pop a stethoscope on someone's chest and for you to hear what the chest sounds like, sounds amazing…she said that my nursing home has it because she's put it there, but I've not used it with them…I don't know if they're not using it because they don't know how to do it, or they don't have the time*” (P3, GP).  “*if it's [the virtual care call] not initiated by the nursing home and their staff don't have training in it, there's no way I can do it*” (P5, GP).  “*I would say, not really [used. Only used] once a week, maybe a couple of times a month*” (P26, RN). |
