# Supplementary material for: Virtual Care and Health Care Access: Pragmatic Evaluation of Implementation, Acceptance, and Use in General Practice and Aged Care Homes
Source: J Med Internet Res. 2026 Jun 12;28:e89019. doi: 10.2196/89019 (PMC13263008; doi:10.2196/89019)
Supplement: Checklist 1 [file jmir-v28-e89019-s004.docx]

**COREQ Checklist**

| Topic | Item No. | Guide Questions | Page No. |
| --- | --- | --- | --- |
| **Domain 1: Research team and reflexivity** | | | |
| *Personal Characteristics* | | | |
| Interviewer/facilitator | 1 | Which author/s conducted the interview or focus group? | 3 |
| Credentials | 2 | What were the researcher’s credentials? *e.g. PhD, MD* | 1, 3 |
| Occupation | 3 | What was their occupation at the time of the study? | 1, 3 |
| Gender | 4 | Was the researcher male or female? | N/A |
| Experience and training | 5 | What experience or training did the researcher have? | 3 |
| *Relationship with participants* | | | |
| Relationship established | 6 | Was a relationship established prior to study commencement? | 3 |
| Participant knowledge of the interviewer | 7 | What did the participants know about the researcher? *e.g. personal goals, reasons for doing the research* | Multimedia Appendix 1 |
| Interviewer characteristics | 8 | What characteristics were reported about the interviewer/facilitator? *e.g. Bias, assumptions, reasons and interests in the research topic* | 3 |
| **Domain 2: Study design** | | | |
| *Theoretical framework* | | | |
| Methodological orientation and Theory | 9 | What methodological orientation was stated to underpin the study? e.g. *grounded theory, discourse analysis, ethnography, phenomenology, content analysis* | 4 |
| *Participant selection* | | | |
| Sampling | 10 | How were participants selected? *e.g. purposive, convenience, consecutive, snowball* | 3 |
| Method of approach | 11 | How were participants approached? *e.g. face-to-face, telephone, mail, email* | 3 |
| Sample size | 12 | How many participants were in the study? | 4 |
| Non-participation | 13 | How many people refused to participate or dropped out? Reasons? | N/A |
| *Setting* | | | |
| Setting of data collection | 14 | Where was the data collected? *e.g. home, clinic, workplace* | 3-4 |
| Presence of non-participants | 15 | Was anyone else present besides the participants and researchers? | - |
| Description of sample | 16 | What are the important characteristics of the sample? *e.g. demographic data* | 4-5  Tables 2 and 3 |
| *Data collection* | | | |
| Interview guide | 17 | Were questions, prompts, guides provided by the authors? Was it pilot tested? | Multimedia Appendix 1 |
| Repeat interviews | 18 | Were repeat interviews carried out? If yes, how many? | N/A |
| Audio/visual recording | 19 | Did the research use audio or visual recording to collect the data? | 3 |
| Field notes | 20 | Were field notes made during and/or after the interview or focus group? | N/A |
| Duration | 21 | What was the duration of the interviews or focus group? | 4 |
| Data saturation | 22 | Was data saturation discussed? | 3-4 |
| Transcripts returned | 23 | Were transcripts returned to participants for comment and/or correction? | N/A |
| **Domain3: Analysis and findings** | | | |
| *Data analysis* | | | |
| Number of data coders | 24 | How many data coders coded the data? | 4 |
| Description of the coding tree | 25 | Did authors provide a description of the coding tree? | - |
| Derivation of themes | 26 | Were themes identified in advance or derived from the data? | 4 |
| Software | 27 | What software, if applicable, was used to manage the data? | 4 |
| Participant checking | 28 | Did participants provide feedback on the findings? | - |
| *Reporting* | | | |
| Quotations presented | 29 | Were participant quotations presented to illustrate the themes/findings? Was each quotation identified? *e.g. participant number* | Multimedia Appendix 2 |
| Data and findings consistent | 30 | Was there consistency between the data presented and the findings? | 5-11 |
| Clarity of major themes | 31 | Were major themes clearly presented in the findings? | 5-11 |
| Clarity of minor themes | 32 | Is there a description of diverse cases or discussion of minor themes? | 5-13 |
